# What Makes Joint Visual Search Efficient? A Distractor Suppression Account

**DOI:** 10.3390/bs16091503

**Published:** 2026-08-27

**Authors:** Xinyue Zhang, Xiaolong Xiang, Mengfei Zhao, Jiahao Lu, Jun Wang

**Affiliations:** 1 School of Psychology, Zhejiang Normal University, Jinhua 321000, China; 2 School of Humanities and Social Science, Gannan Medical University, Ganzhou 341000, China; 3 Zhejiang Philosophy and Social Science Laboratory for the Mental Health and Crisis Intervention of Children and Adolescents, Zhejiang Normal University, Jinhua 321000, China

**Keywords:** joint visual search, distractor suppression, priority map, joint action

## Abstract

Visual search is thought to operate via a priority map that weights targets against distractors. Any process that modulates the weight assigned to distractors can influence search efficiency. Prior work on joint visual search has examined how distractors matching a partner’s target receive enhanced weighting and impair individual performance. The present study asks instead whether the items the partner must reject are processed more efficiently in a joint context, thereby improving individual search efficiency. One possible account is enhanced distractor suppression, on which such items are down-weighted more effectively when another person is rejecting them concurrently. Participants searched shared multi-item displays for color-defined targets, and search slopes (response time per item) were analyzed with linear mixed-effects models. Experiment 1 (*N* = 114) compared joint, individual, and passive observer conditions. Experiment 2 (*N* = 76) compared in-group and out-group pairs formed via a minimal-group paradigm. In Experiment 1 the joint slope was shallower than both the individual and passive observer slopes. In Experiment 2 the slope was shallower for out-group than for in-group pairs, revealing a group-based modulation of joint search efficiency. On log-transformed response times, this difference appeared instead as a constant proportional advantage. Although search slopes index overall efficiency rather than directly measuring suppression, and alternative processes could also contribute to shallower slopes, these findings are consistent with a distractor suppression account of what makes joint visual search efficient.

## 1. Introduction

In everyday life, humans constantly search for relevant information in complex visual environments. To do so efficiently, the visual system must direct attention toward the most relevant items. The Guided Search 6.0 model (Wolfe, 2021) offers a useful framework for understanding how this is achieved. Within Guided Search 6.0, attention is guided by a priority map, a continuously updated representation that integrates bottom-up salience with top-down task goals to determine where attention should move next. Search efficiency depends on how strongly targets are prioritized over distractors in this map. The search slope, which is the increase in response time per additional display item, provides an interpretable index of this balance. Each added distractor imposes an attentional cost on target selection, and any process that lowers distractor weights should flatten the slope.

While most supporting evidence for the priority map hypothesis comes from individual visual search, recent work has begun to show that the joint search context is another important factor that modulates attentional weighting in the priority map, sometimes facilitating search and sometimes interfering with it. When partners search for the same target, search efficiency reliably improves across a range of collaborative search and coordination tasks (Neider et al., 2010; Dale et al., 2011; Wahn et al., 2018; Niehorster et al., 2019; Wahn & Schmitz, 2023). Two findings suggest that this benefit reflects a redistribution of attentional weights rather than a general boost in motivation or arousal. First, the benefit is strongest when the task is difficult and requires exhaustive search (H. Zhang et al., 2025), precisely when distractor management is most demanding. Second, shared search goals produce a systematic shift in visual weighting, with attention drawn toward items that are salient for the partner (Richardson et al., 2012). When partners pursue independent goals, however, joint search can instead produce interference. In the participant’s priority map, the partner’s target gains weight through its acquired social salience, drawing attention away from the participant’s own target and slowing search relative to individual search (Dötsch et al., 2022). Co-representation theory offers a unified account of both patterns. On this view, people automatically and implicitly represent a co-actor’s actions, so that an action assigned to another person is represented in much the same way as an action assigned to oneself (Knoblich & Sebanz, 2006). For example, individuals do represent a wide range of information about their co-actors, including stimulus–response mappings (Sebanz et al., 2003, 2005), statistical properties of stimuli (Zheng & Wang, 2024b), task regularities (Zheng & Wang, 2023, 2024a; Zhao et al., 2025), and even relatively stable attributes such as personality traits (Pan et al., 2026). Under shared goals, co-representing the partner’s target supports coordinated search. Under independent goals, the same mechanism instead assigns weight to the partner’s irrelevant target and produces a cost.

The research on joint search reviewed above has focused almost exclusively on how a co-represented partner’s target affects performance. Yet efficient search depends just as much on the suppression of distractors, which is a functionally independent process rather than a byproduct of target selection (Sawaki & Luck, 2010). Distractor suppression spans multiple mechanisms, from passive filtering to experience-based learned suppression and goal-driven strategic suppression, the latter forms being active and trainable rather than fixed (Theeuwes, 2025). Critically, whether a stimulus captures or is suppressed is governed by its relevance to the current task rather than by physical salience alone (Y. Zhang & Gaspelin, 2024). Because suppression of this kind is set by what is currently relevant rather than fixed in advance, it may also be sensitive to the presence of a co-actor searching the same display. Joint-action research offers a precedent for such an influence at the level of inhibition rather than selection. In social inhibition of return, responses are slower to a location that a partner has just acted upon, much as they are to a location one has acted upon oneself (Welsh et al., 2005), indicating that a partner’s task-relevant behavior can shape the searcher’s own attentional inhibition. Having the partner concurrently reject the nontarget items in a shared display could therefore alter how those items are processed. We therefore ask whether such items are processed more efficiently in a joint context than when the same display is searched alone, and we frame this as a distractor suppression account of joint search performance. Existing findings raise this possibility without settling it. When two people search a shared display for differently colored targets, they fail to learn the repeated spatial configuration of the partner-colored nontarget items (Sakata et al., 2021). Effective suppression of these items has been proposed as one possible account of this finding (Xie et al., 2025), suggesting that joint search may influence the processing of task-irrelevant information.

To test this prediction, we adapted an established visual search paradigm (Wolfe, 2020) to a joint-action context. In Experiment 1, participants searched under one of three conditions. In the joint condition, two participants searched the same display simultaneously, each for their own color-defined target. In the individual condition, one person searched alone. In the passive observer condition, an active searcher was accompanied by a non-searching partner, allowing us to hold social presence constant. If joint search enhances the rejection of nontarget items, the joint condition should yield the shallowest slope, shallower than both the individual and passive observer conditions. Experiment 1 asks whether a joint search advantage exists, and Experiment 2 asks whether it depends on who the partner is. Experiment 2 manipulated the social relationship between partners while holding the display and task constant, pairing participants with an in-group or out-group member through a minimal-group paradigm (McClung et al., 2013). Two accounts make opposing predictions here. If the joint advantage depends on taking up the partner’s concurrent task, then it should be larger for in-group pairs, because a partner’s task is co-represented more readily when the partner is an in-group member (McClung et al., 2013; Müller et al., 2011). If instead the advantage depends on how readily partner-related items can be down-weighted, then it should be larger for out-group pairs, because in-group membership increases self–other overlap (Enock et al., 2020; Aron et al., 2022) and self-associated items resist attentional suppression (Sui et al., 2015). Because these accounts point in opposite directions, we made no directional prediction and treat the outcome as informative about which of the two better describes joint search.

## 2. Experiment 1

### 2.1. Method

#### 2.1.1. Participants

Experiment 1 included 114 students from Zhejiang Normal University (*M* = 22.02 years, *SD* = 2.00, 17 male, 97 female; 3 males in the individual condition, 2 in the joint condition, and 12 in the passive observer condition). Sample size was determined a priori with G*Power version 3.1.9.7 (Heinrich-Heine-Universität Düsseldorf, Düsseldorf, Germany; Faul et al., 2009) for a fixed-effects one-way analysis of variance comparing the between-participants conditions, assuming an effect size of *f* = 0.25, *α* = 0.05, and power = 0.85. We recruited 114 participants to accommodate the three experimental conditions and paired testing. This calculation is retained for transparency, but it does not reflect the mixed-effects structure of the design or the clustering of participants within units. We additionally report a simulation-based sensitivity analysis that takes the mixed-effects structure and the clustering of participants within units into account. Power was assessed separately for the two focal contrasts involving the joint condition, using the realized sample structure of 95 independent units. For the joint versus individual slope difference, power was 0.86 for a difference of 1.5 ms per item, 0.50 for 1.0 ms per item, and 0.19 for 0.5 ms per item. For the joint versus passive observer difference, the corresponding values were 0.87, 0.54, and 0.14. In both cases the smallest difference detectable at 80% power was approximately 1.5 ms per item.

All participants were right-handed, had normal or corrected-to-normal vision, and were neither color-blind nor color-weak. Written informed consent was obtained in accordance with the Declaration of Helsinki. Participants were compensated for their time, and the study was approved by the Ethics Committee of Zhejiang Normal University.

#### 2.1.2. Apparatus and Stimuli

Following a classic visual search paradigm (Wolfe, 2020), we created L and T figures, each subtending 300 pixels. Each display contained two color-defined subsets, purple (RGB = 238, 84, 221) and green (RGB = 52, 182, 81), presented together on a gray background (RGB = 98, 98, 104). Set size, defined as the total number of items across both colors, was 6, 12, 18, 24, 30, 36, or 42, divided equally between the two subsets. Each subset contained exactly one T among the remaining Ls. Every L was therefore a nontarget for both searchers, whereas each T was the target for one searcher and a nontarget for the other. Items were arranged randomly within an 11 × 10 grid, with adjacent item centers separated by 2° to prevent clustering, and each item’s orientation was drawn randomly from 0°, 90°, 180°, to 270°. Which of the two colors defined a given searcher’s target was counterbalanced within pairs so that the two members of a pair always searched for targets of different colors, and each color served as the target color equally often for the left-seated and the right-seated positions.

**Figure 1:**
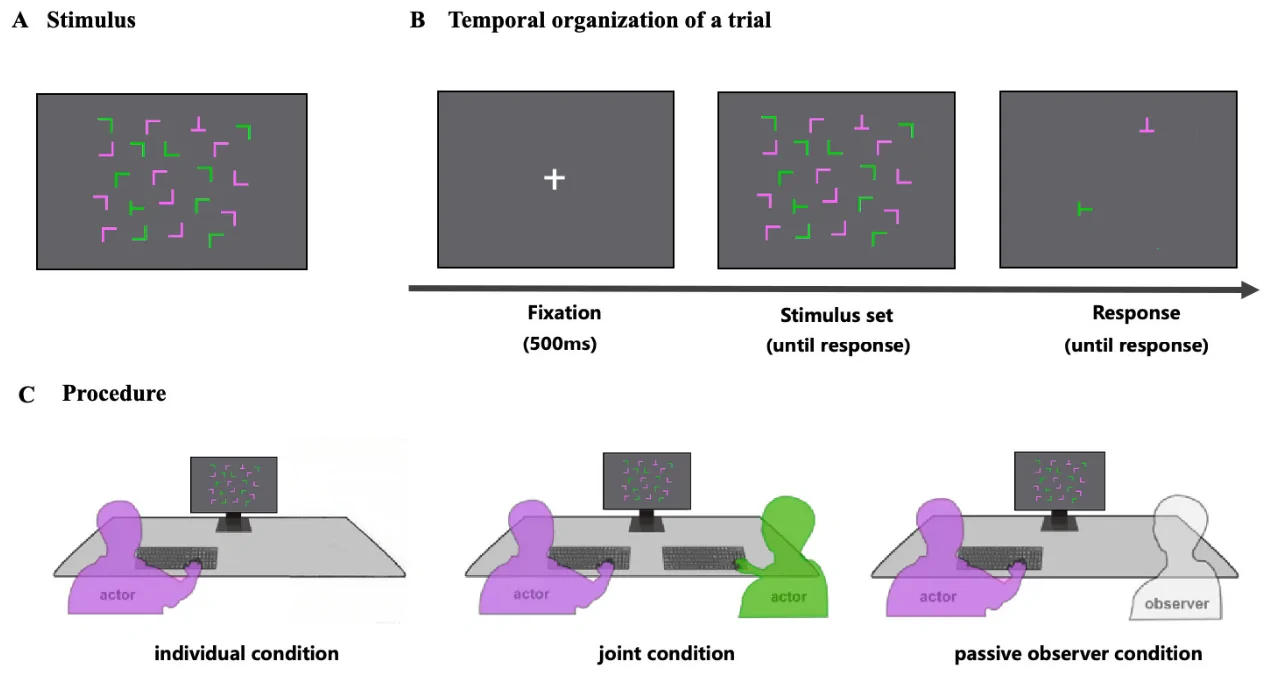
Overview of the procedure of Experiment 1: (**A**) Each display contained 6, 12, 18, 24, 30, 36, or 42 L and T figures arranged in an 11 × 10 grid. The items formed two equally sized color subsets, purple and green, each containing a single T among the remaining Ls, so that every display held two Ts in total, one per color. The orientation of each item was randomly assigned to one of four angles, 0°, 90°, 180°, or 270°. (**B**) Each trial began with a 500 ms fixation point, followed by the display at the center of the screen until a keyboard response was made, after which the next trial began. Participants judged the orientation of their assigned target and responded with the corresponding key (S/D/W/A for the left-seated participant, K/L/I/J for the right-seated participant). The white cross denotes the fixation point, and the horizontal arrow below the panels indicates the temporal progression of a trial. (**C**) Schematic figures represent participants. In the joint condition, two participants sat side by side and searched the same display, each responding only to their pre-assigned target (here, the left-seated participant responds to the purple T). In the individual condition, a single participant was seated at either the left or right position and responded to their pre-assigned target (here, a left-seated participant responds to the purple T). In the passive observer condition, a participant searched the display while a second person was seated beside them and did not respond. The color of each schematic figure indicates the target color assigned to that participant, and the grey figure denotes the passive observer.

Testing took place in a quiet, dimly lit room (0.35 cd/m^2^). Event scheduling and response recording were controlled by custom code written in MATLAB R2025b (The MathWorks Inc., Natick, MA, USA) with Psychtoolbox-3 (Brainard, 1997). Stimuli were presented on a 27-inch ASUS ROG Swift PG279Q LCD monitor (ASUSTeK Computer Inc., Taipei, China) running at a desktop resolution of 1920 × 1080 pixels with a refresh rate of 120 Hz, and each participant sat 60 cm from the screen (Figure 1).

#### 2.1.3. Procedure and Design

Participants were randomly assigned to one of three conditions, with 38 participants per condition. Pairing was restricted to same-sex dyads of strangers, a restriction adopted to hold constant any differences in social interaction that mixed-sex pairing might introduce. Seat position and the color of the assigned target were counterbalanced so that participants across the left-seated and right-seated positions were equally often paired with each target color. In the joint condition, 19 pairs sat side by side, and each member searched for their own pre-assigned target; for example, the left-seated participant searched for purple Ts. In the passive observer condition, each of the 38 participants searched while seated beside a confederate from the research team, who was present throughout but performed no task and made no responses, so every participant in this condition contributed search data and constituted an independent unit. In the individual condition, 38 participants each sat alone at either the left or the right position and searched for their pre-assigned target; for example, a right-seated participant searched for green Ts.

In all conditions, each trial began with a 500 ms fixation cross, followed by the display at the center of the screen, and the next trial began only after all active participants had responded. Participants judged the orientation of their assigned target (0°, 90°, 180°, or 270°) by keypress on QWERTY keyboards. Left-seated participants used the S, D, W, and A keys (corresponding to 0°, 90°, 180°, and 270°, respectively), and right-seated participants used the K, L, I, and J keys. In the individual condition, the single participant used the key set corresponding to their seated position. The two members of a pair used separate keyboards placed side by side. The display remained unchanged until all active participants had responded, so a partner’s response produced no visible change to the display, and no feedback about a partner’s accuracy or speed was given at any point.

Following task instructions, participants completed 14 practice trials, with each of the seven set sizes presented twice. The main experiment comprised 252 trials, with each set size presented 36 times, organized into blocks of 36 with a rest break between blocks.

#### 2.1.4. Data Analyses

All analyses were conducted in R version 4.6.0 (R Foundation for Statistical Computing, Vienna, Austria; R Core Team, 2026). Data were trimmed in three sequential steps. First, trials with response times below 200 ms or with incorrect responses were removed (1%). Second, for each participant and set size, trials with response times exceeding ±2.5 *SD* from that participant’s mean for the corresponding set size were excluded (2.38%). Third, trials with response times exceeding ±3 *SD* from the grand mean for the corresponding set size were removed (0.94%).

Because observations within dyads are not independent, we analyzed response times with linear mixed models (LMMs), following procedures from prior joint-action research (Zheng & Wang, 2024b). Set size was entered as a within-subjects predictor, condition (individual, joint, observer) as a between-subjects predictor, and their interaction as fixed effects. The model included random intercepts for participants nested within dyads. In the joint condition a dyad comprised the two participants tested together, giving 19 dyads. In the individual and passive observer conditions each participant contributed as a single independent unit, giving 38 units in each condition and 95 units in total. Variance components and a comparison against a model without the unit level are reported in the Supplementary Materials. Retention after trimming was 95.0% of trials in the individual condition and 96.0% in both the joint and passive observer conditions. To confirm that the nesting did not drive the results, we refitted the models with a single random intercept for participants and obtained fixed effects that were unchanged in sign, magnitude, and significance. Significant interactions were followed up with simple slope analyses.

*RT* ~ *set_size* × *condition* + (1|*dyad*/*subject_id*)

Models were estimated with the lme4 package version 2.0.1 (Bates et al., 2015). Main and interaction effects were tested with the anova function using Satterthwaite-corrected *p*-values from the lmerTest package version 3.2.1 (Kuznetsova et al., 2017). Post hoc contrasts and simple slope analyses used the emmeans version 2.0.3 (Lenth & Piaskowski, 2026) and interactions packages version 1.2.0 (Long, 2024). Trial exclusions at each trimming step, broken down by condition and set size, model diagnostics for the response time and accuracy models, and the influential-case analysis based on Cook’s distance are reported in the Supplementary Materials, together with the analysis scripts and the trial-level data listed in the Data Availability Statement.

### 2.2. Results

#### 2.2.1. Response Times

Table 1 presents the LMM results for Experiment 1. The main effect of condition on baseline RT was not significant. Relative to the individual condition, neither the joint condition (*β* = −13.46, *SE* = 32.41, *t*(104.09) = −0.42, *p* = 0.679) nor the passive observer condition (*β* = 33.44, *SE* = 28.60, *t*(142.93) = 1.17, *p* = 0.244) differed after accounting for set size. The main effect of set size was significant (*β* = 15.95, *SE* = 0.35, *t*(681) = 45.67, *p* < 0.001), confirming that RTs increased with display size. Critically, the omnibus Type III test revealed a significant condition-by-set-size interaction, *F*(2, 681) = 19.75, *p* < 0.001, with no main effect of condition, *F*(2, 118) = 1.22, *p* = 0.298, indicating that the rate at which RTs increased with display size differed across conditions. We decomposed this interaction with simple slope analyses, reported below.

**Figure 2:**
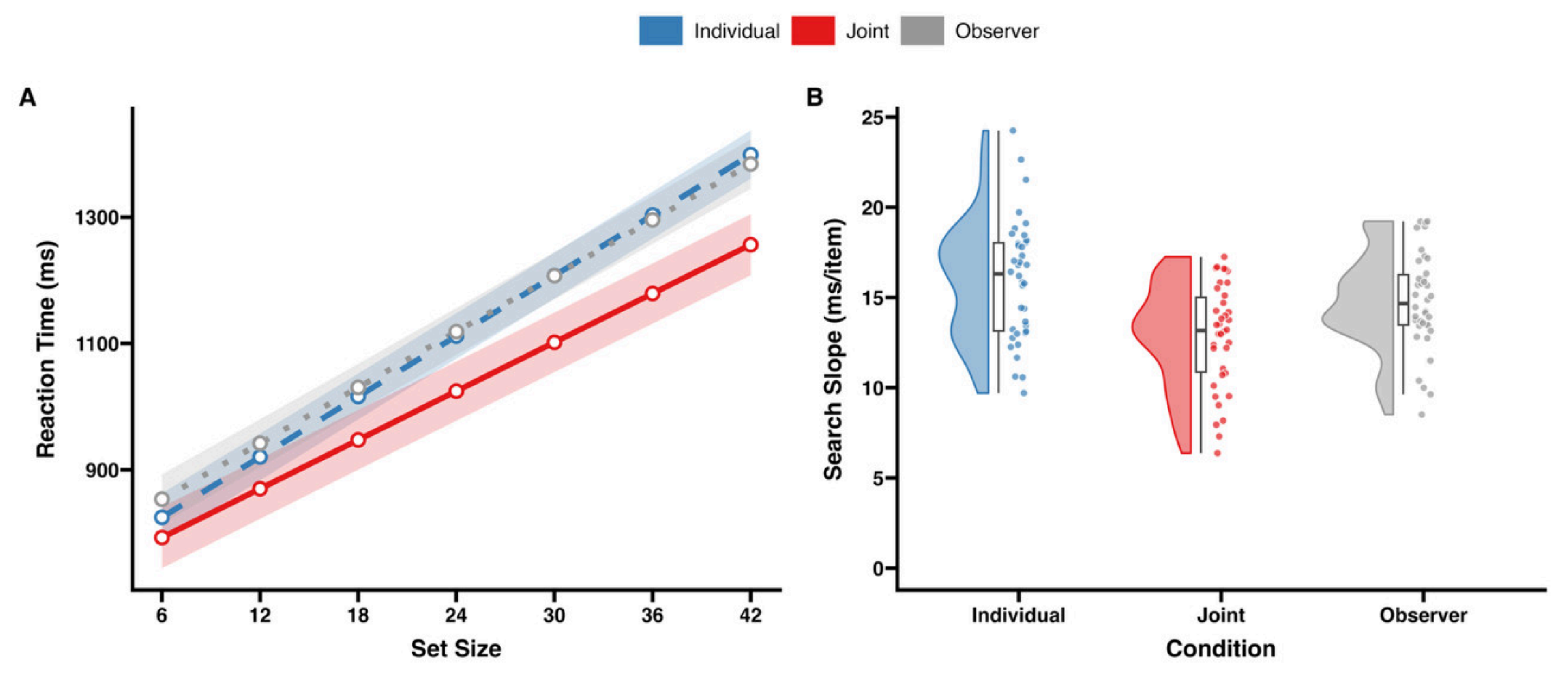
(**A**) Simple slopes of set size on response time across the three conditions, with shaded bands showing 95% confidence intervals. (**B**) Distribution of participant-level search slopes in each condition, with individual participants shown as points and the condition mean and its 95% confidence interval overlaid.

To examine how condition modulated the effect of set size on RTs, we conducted simple slope analyses with the interactions package in R (Long, 2024), with pairwise comparisons of the slopes computed using emmeans (Lenth & Piaskowski, 2026). The set-size slope was 15.95 ms/item in the individual condition (*SE* = 0.35), 12.87 ms/item in the joint condition (*SE* = 0.35), and 14.79 ms/item in the passive observer condition (*SE* = 0.35), each significantly greater than zero (all *p*s < 0.001). All pairwise slope contrasts are Tukey-adjusted. The joint slope was significantly shallower than both the individual slope (difference = −3.07 ms/item, *SE* = 0.49, *t*(681) = −6.22, *p* < 0.001) and the passive observer slope (difference = −1.91 ms/item, *SE* = 0.49, *t*(681) = −3.87, *p* < 0.001). The comparison between the passive observer and the individual condition was significant under Tukey adjustment in this specification (difference = −1.16 ms/item, *SE* = 0.49, *t*(681) = −2.35, *p* = 0.0498). Because set size varied within participants, we also fitted a model with by-participant and by-dyad random slopes for set size, which was favored by model comparison, χ^2^(4) = 70.77, *p* < 0.001. Under this specification the interaction remained significant, *F*(2, 100.36) = 9.78, *p* < 0.001, as did the joint versus individual difference (*p* < 0.001) and the joint versus passive observer difference (*p* = 0.022), whereas the non-focal comparison between the passive observer and individual conditions did not (*p* = 0.213). This random-slope model produced a boundary singular fit, so the random-intercept model is retained as the primary specification, in line with recommendations to prefer parsimonious random-effects structures when the maximal structure is not supported by the data (Matuschek et al., 2017). We therefore treat the advantage of joint search over both individual search and passive observation as robust, and we draw no conclusion from the comparison between the passive observer and individual conditions, for which the design was not powered (see Figure 2).

We also report the model with set size mean-centered at 24 items, the average observed value, so that the intercept refers to a set size that was actually presented. Predicted RT at that set size was 1111.77 ms, and the joint condition was 87.22 ms faster than the individual condition (*SE* = 30.16, *t*(78.32) = −2.89, *p* = 0.005), whereas the passive observer condition did not differ from the individual condition (*β* = 5.58, *SE* = 26.02, *p* = 0.831). In relative terms, the joint slope was 19.3% shallower than the individual slope, corresponding to an advantage of 129 ms at the largest set size, and the passive observer slope was 7.3% shallower, corresponding to 49 ms. Means, standard deviations, and 95% confidence intervals for every condition-by-set-size cell are reported in the Supplementary Materials, and the distribution of participant-level search slopes is shown in panel B of Figure 2. Their means coincide with the model estimates, with standard deviations of 3.36, 2.88, and 2.69, respectively.

Four further robustness checks were applied. The interaction was larger with minimally trimmed data in which only errors and responses faster than 200 ms were removed, *β* = −4.78, *SE* = 0.56, *p* < 0.001. The interaction also remained significant on log-transformed response times, *β* = −0.0019, *SE* = 0.0005, *p* < 0.001, indicating that the joint advantage is not reducible to a proportional speedup (Supplementary Table S5). Because the random-slope model above produced a singular fit, we also fitted two simplified random-slope structures, one omitting the intercept–slope correlations and one retaining slopes at the participant level only. Both converged without a singular fit and were preferred to the random-intercept model on AIC (9280.3 and 9280.4 against 9344.2), and the joint versus individual difference remained significant in each, *t*(70.43) = −4.16, *p* < 0.001 and *t*(111) = −4.48, *p* < 0.001, as did the joint versus passive observer difference, *p* = 0.031 and *p* = 0.017. Eight of the 95 independent units exceeded the conventional Cook’s distance threshold of 4/*n* = 0.042; excluding them attenuated the effects without removing them, with the joint versus individual difference falling from 3.07 to 2.19 ms/item, *t*(609) = 4.35, *p* < 0.001, and the joint versus passive observer difference from 1.91 to 1.50 ms/item, *t*(609) = 3.03, *p* = 0.007. Full output for these checks is reported in the Supplementary Materials.

#### 2.2.2. Accuracy

Mean accuracy, computed on all trials before trimming, was high in all conditions, 99.33% (individual), 98.74% (joint), and 99.24% (passive observer), ranging from 94.05% to 100% with no systematic trends. Accuracy was modeled with a GLMM (binomial family) using the same fixed- and random-effects structure as the RT model, with random intercepts for participants nested within dyads. The model was fitted to all trials surviving the response-time-trimming steps, with errors retained as the outcome. The model revealed a statistically significant but numerically small main effect of condition (joint vs. individual: *β* = −0.90, *SE* = 0.41, *z* = −2.18, *p* = 0.030). Crucially, this difference did not vary with set size, as there was no main effect of set size and no condition-by-set-size interaction (all *p*s > 0.05). A speed–accuracy trade-off of the kind that could produce the slope effect would require the accuracy difference to grow with set size, and it was instead constant, which makes a size-dependent trade-off an unlikely account of the slope difference. It does not exclude a constant shift in response criterion or a general difference in speed–accuracy strategy between conditions, both of which would be expected to affect overall RT rather than its dependence on set size. The complete GLMM output, with coefficients, standard errors, z values, *p* values, and 95% confidence intervals for every term, is reported in the Supplementary Materials.

In summary, response times increased with set size in all conditions, but the slope was shallowest in the joint condition, significantly shallower than in both the individual and the passive observer conditions. The joint advantage therefore cannot be attributed to the presence of another person alone, and we consider more effective rejection of the nontarget items as one interpretation of this additional component, which only an actively searching partner affords. Accuracy was near the ceiling throughout and did not vary with set size.

## 3. Experiment 2

Experiment 1 established that the search slope was shallowest in the joint condition. Given the growing body of literature suggesting that the social relationship between co-actors influences how their tasks interact, Experiment 2 examined whether this difference in search efficiency depends on that relationship. To this end, we manipulated the relationship between the co-actors while keeping the display and the task constant.

### 3.1. Method

#### 3.1.1. Participants

Seventy-six new undergraduate students (*M* = 21.24 years, *SD* = 2.20, 12 male, 64 female; six males in the in-group condition, six in the out-group condition) took part in Experiment 2. They were recruited and randomly paired following the same procedure as the joint condition of Experiment 1. As in Experiment 1, we report a simulation-based sensitivity analysis that takes the clustering of participants within dyads into account. With the realized structure of 38 dyads, power for the condition-by-set-size interaction was 0.90 for an effect of 1.5 ms per item, 0.61 for 1.0 ms per item, and 0.20 for 0.5 ms per item.

#### 3.1.2. Apparatus and Stimuli

The apparatus and stimuli were identical to those used in the joint condition of Experiment 1.

#### 3.1.3. Procedure and Design

On arrival, participants completed the Big Five Inventory-2 (BFI-2; B. Zhang et al., 2022), following previous studies that used the minimal-group paradigm (Simpson & Todd, 2017; Hutchings et al., 2021). The experimenter ostensibly scored their responses and assigned each participant to either an “orange” or a “green” personality type, although the assignment was in fact random. To reinforce group identity, participants received a colored wristband and vest corresponding to their assigned type (e.g., orange for the orange type) and were instructed to wear them for the remainder of the session as a reminder of their group membership. Participants were then paired, subject to the same-sex and stranger restrictions used in Experiment 1, and each pair was randomly assigned to receive either the same label for both members, constituting an in-group dyad, or different labels, constituting an out-group dyad, with 19 dyads in each condition. Seat position and target color were counterbalanced as in Experiment 1. Participants then completed the joint visual search task, following the same procedure as the joint condition of Experiment 1. The experimenter was not blind to condition. The condition was determined before testing began, instructions were standardized, and the dependent measures were recorded automatically by the computer. The deceptive personality feedback used to create the minimal groups was reviewed and specifically approved by the Ethics Committee of Zhejiang Normal University as part of the study protocol. At the end of the session, participants were asked whether they had doubted the personality classification or the group assignment, and no participant reported such doubts. All participants were then fully debriefed about the deception and about the random basis of the group assignment.

#### 3.1.4. Data Analyses

Data were trimmed and analyzed using the same procedures as in Experiment 1. Trials with response times below 200 ms or with incorrect responses (0.96%), trials exceeding ±2.5 SD of each participant’s mean for the corresponding set size (2.67%), and trials exceeding ±3 SD of the grand mean for the corresponding set size (1.05%) were excluded. Response times were modeled with the same linear mixed model as Experiment 1, *RT* ~ *set_size* × *condition* + (1|*dyad*/*subject_id*), with condition (in-group, out-group) as a between-subjects predictor and random intercepts for participants nested within dyads. In Experiment 2 all participants were tested in pairs, and a dyad comprised the two participants tested together, giving 38 dyads and 76 participants. Retention after trimming was 95.1% of trials in the in-group condition and 95.6% in the out-group condition, and no participant lost all of their trials. As in Experiment 1, trial exclusions by condition and set size, variance components and the intracluster correlation, model diagnostics, and the influential-case analysis are reported in the Supplementary Materials.

### 3.2. Results

#### 3.2.1. Manipulation Check

After the task, participants rated their perceived similarity to their partner on a 7-point scale (1 = not at all similar, 7 = extremely similar). As predicted, in-group pairs reported greater similarity (*M* = 5.58, *SD* = 1.11) than out-group pairs (*M* = 3.95, *SD* = 0.64), *t*(59.16) = 7.82, *p* < 0.001, *d* = 1.79. Because participants were nested within dyads, we also analyzed these ratings with dyad-aware models. Aggregating to a single value per dyad gave the same conclusion, *t*(26.93) = 6.42, *p* < 0.001, as did a linear mixed model with random intercepts for dyad, *β* = −1.63, *SE* = 0.25, *t*(36) = −6.42, *p* < 0.001. Together with the absence of reported suspicion, this indicates that the manipulation shifted how participants viewed their partner.

#### 3.2.2. Response Times

Table 2 presents the LMM results for Experiment 2. The main effect of set size was significant (*β* = 12.67, *SE* = 0.32, *t*(454.00) = 39.48, *p* < 0.001), confirming that RTs increased with display size. The main effect of condition was also significant (*β* = −55.54, *SE* = 25.94, *t*(52.88) = −2.14, *p* = 0.037), with shorter baseline RTs in the out-group condition. Critically, the condition-by-set-size interaction was significant (*β* = −1.56, *SE* = 0.45, *t*(454.00) = −3.45, *p* < 0.001). The omnibus Type III tests confirmed the same pattern, with a significant condition-by-set-size interaction, *F*(1, 454) = 11.87, *p* < 0.001, alongside effects of set size, *F*(1, 454) = 2744.20, *p* < 0.001, and condition, *F*(1, 52.88) = 4.58, *p* = 0.037. The set-size slope was significantly shallower in the out-group condition, indicating a more efficient search for out-group pairs.

**Figure 3:**
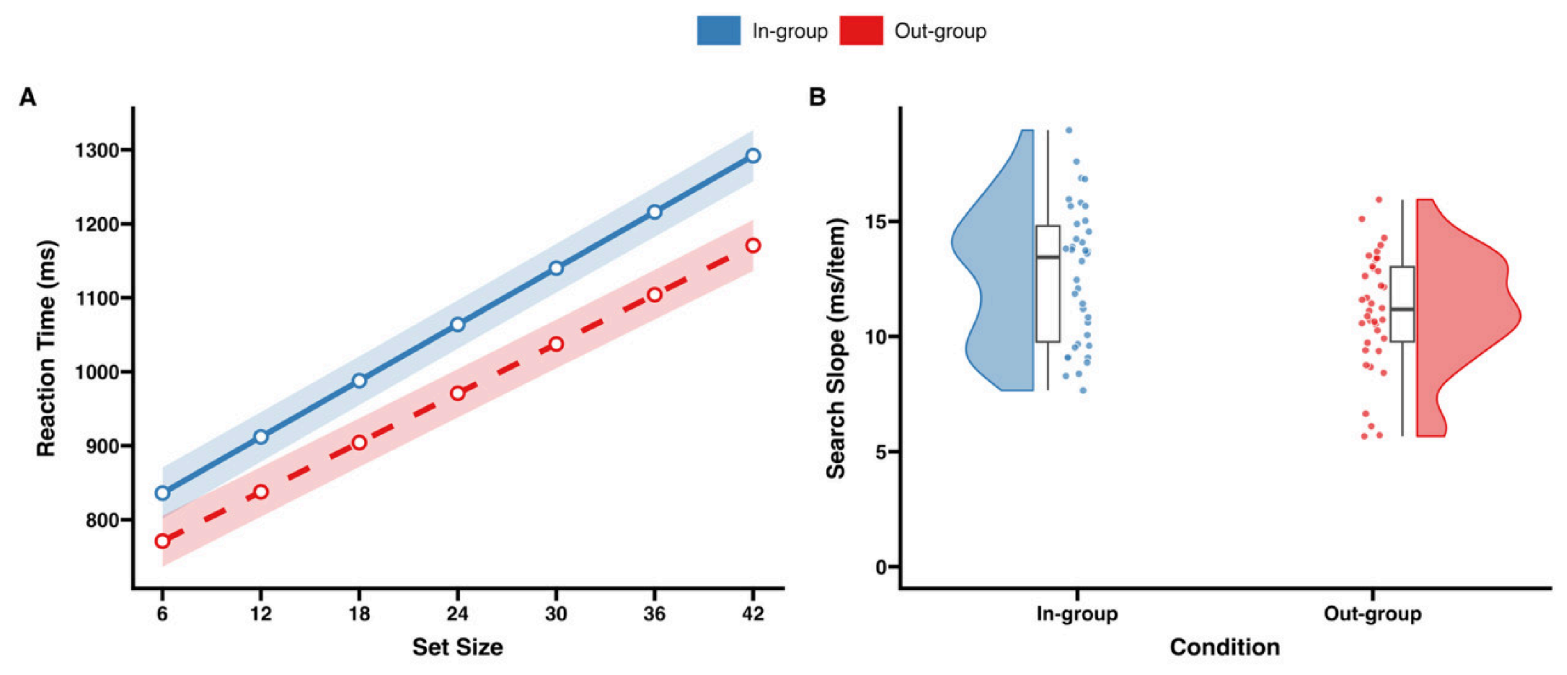
(**A**) Simple slopes of set size on response time across the two conditions, with shaded bands showing 95% confidence intervals. (**B**) Distribution of participant-level search slopes in each condition, with individual participants shown as points and the condition mean and its 95% confidence interval overlaid.

To examine how condition modulated the effect of set size on RTs, we conducted simple slope analyses with the interactions package in R (Long, 2024). The slope of set size on RTs was 12.67 ms/item in the in-group condition (*SE* = 0.32) and 11.11 ms/item in the out-group condition (*SE* = 0.32). This difference was significant (difference = 1.56 ms/item, *SE* = 0.45, *t*(454) = 3.45, *p* < 0.001), with the shallower out-group slope indicating more efficient search for out-group pairs (see Figure 3).

With set size mean-centered at 24 items, the average observed value and predicted RT were 1064.00 ms, and out-group pairs were 93.08 ms faster than in-group pairs (*SE* = 23.55, *t*(36) = −3.95, *p* < 0.001). In relative terms, the out-group slope was 12.3% shallower than the in-group slope, corresponding to an advantage of 66 ms at the largest set size. Means, standard deviations, and 95% confidence intervals for every condition-by-set-size cell are reported in the Supplementary Materials, and the distribution of participant-level search slopes is shown in panel B of Figure 3. Slopes fitted separately for each participant averaged 12.73 ms/item for in-group pairs (*SD* = 2.97) and 11.10 ms/item for out-group pairs (*SD* = 2.52).

We examined the robustness of this pattern in five ways. A model with by-participant and by-dyad random slopes for set size was favored by model comparison, χ^2^(4) = 57.64, *p* < 0.001, and the interaction remained significant under it, *F*(1, 43.55) = 5.18, *p* = 0.028. Because that model produced a singular fit, we also fitted two simplified random-slope structures, one omitting the intercept–slope correlations and one retaining slopes at the participant level only. Both converged without a singular fit and were preferred to the random-intercept model on AIC (6068.1 and 6068.7 against 6117.0), and the interaction remained significant in each, *t*(39.67) = −2.16, *p* = 0.037 and *t*(74) = −2.48, *p* = 0.016. The random-intercept model is retained as the primary specification. Six of the 38 dyads exceeded the conventional Cook’s distance threshold of 4/*n* = 0.105; excluding them reduced the interaction from −1.56 to −1.13 ms/item while leaving it significant, *SE* = 0.48, *t*(382) = −2.36, *p* = 0.019. Full output for these checks is reported in the Supplementary Materials. The interaction was also significant on minimally trimmed data, *β* = −1.65, *SE* = 0.51, *p* = 0.001. On log-transformed response times, the interaction was not significant, *β* = −0.0004, *SE* = 0.0004, *p* = 0.342, whereas the main effect of condition remained, *p* = 0.002, corresponding to a constant proportional advantage of 7.6% for out-group pairs at every set size (Supplementary Table S5). The group-related difference therefore held across every random-effects structure we examined and across both trimming procedures.

#### 3.2.3. Accuracy

Mean accuracy, computed on all trials before trimming, was 99.24% in the in-group condition and 98.87% in the out-group condition, ranging from 96.43% to 100%. Modelled with the same GLMM as in Experiment 1, accuracy showed a statistically significant but numerically small main effect of condition (*β* = −1.05, *SE* = 0.44, *z* = −2.37, *p* = 0.018) that did not vary with set size (set size: *z* = −0.41, *p* = 0.684; interaction: *z* = 1.48, *p* = 0.138). As in Experiment 1, the difference was constant across set sizes, which makes a size-dependent trade-off an unlikely account of the slope effect while leaving open a constant difference in response criterion. Accuracy was again close to the ceiling. The complete GLMM output is reported in the Supplementary Materials.

In summary, response times increased with set size in both conditions, but more steeply for in-group than for out-group pairs, indicating that out-group dyads searched more efficiently. Out-group pairs also had a lower intercept, indicating that they were faster by approximately 55 ms independently of set size; at the mean set size of 24 items, the total advantage was 93 ms. Group membership was therefore associated with a difference in joint search efficiency. Because both conditions involved joint search and Experiment 2 included no individual or observer baseline, this result establishes a difference between two joint conditions rather than a difference in the size of any advantage over searching alone.

## 4. Discussion

The present study asked whether searching alongside a partner concurrently rejecting the nontarget items is associated with greater search efficiency. Experiment 1 showed that the search slope was shallowest in the joint condition, shallower than in both the individual and passive observer conditions. Experiment 2 showed that the slope also differed with the social relationship between partners, being shallower for out-group than for in-group pairs. Search efficiency in joint search therefore depends in part on who the partner is.

The design of Experiment 1 narrows the range of explanations for the joint advantage. First, every condition permitted ordinary color-based filtering of the partner’s subset (Wolfe, 2021), so feature filtering alone is common to all three and cannot explain why the joint slope was the shallowest. Second, the two searchers had different targets and neither could respond on the other’s behalf, so the task could not be divided in the sense of one searcher covering part of the display on the other’s behalf. A strategic allocation of attention across the two color-defined subsets was available in every condition since the same two-color display was used throughout and so falls under the first point rather than being specific to a joint search. Third, the passive observer condition holds social presence constant, and the joint slope was shallower than the observer slope under both the random-intercept and the random-slope specifications. What remains specific to the joint condition is that another person was searching the same display at the same time. The partner did not reject the other color subset as a whole, because that subset contained the partner’s own target. What the partner rejected was the nontarget Ls, which are also the items the searcher had to reject. The joint condition is therefore the only condition in which the items that drive the slope were being rejected by two people at once.

A similar pattern has been reported in joint action research (Zheng & Wang, 2024b), where a co-actor’s stimulus set, normally filtered out as irrelevant, came to bias perception once joint engagement rendered it consequential. More generally, a co-actor’s task can change how an otherwise irrelevant subset is processed, with the direction depending on the role that subset plays in the co-actor’s task. When that subset contains the co-actor’s target, joint engagement favors encoding it rather than suppressing it (Zang et al., 2022). When it consists instead of items the searcher must reject, as in the present study, the observed pattern is consistent with suppression.

The suppression account is not the only explanation for the joint advantage, however. Other possibilities include co-representation of the partner’s target, a general increase in task engagement when someone else is working alongside, and monitoring of the partner’s progress. Coordination of responses between the two searchers can be set aside. Restricting the joint condition to first responses left the advantage over individual search intact, and the group difference in Experiment 2 held within both the first-response and the second-response subsets. The interval between the two responses also grew with set size rather than shrinking, which is the opposite of what convergence on a common response time would produce, and the two subsets fell symmetrically about the joint slope, as selection on response speed alone predicts. These analyses are reported in the Supplementary Materials. A shallower slope shows that the cost of each additional item was lower in joint search, but it does not identify what lowered it. We therefore offer enhanced rejection of nontarget items as one interpretation of this pattern rather than as a demonstrated mechanism, and we regard distinguishing among the remaining accounts as the main task for future work.

These findings can be related to the Guided Search framework. In Guided Search, the priority map weights targets against distractors on the basis of an individual’s own bottom-up salience and top-down goals (Wolfe, 2021), and distractor down-weighting is treated as a process internal to the searcher (Gaspelin & Luck, 2018). The present results raise the possibility of a further source of these weights, namely a co-actor’s concurrent task. On this reading, items that two people are rejecting at the same time would lose priority in the searcher’s map beyond the level reached alone, which would flatten the slope. These results are compatible with the possibility that the priority map is not a strictly individual computation and that it can be shaped in part by what another person is doing. Whereas previous work has reported effects on the target side of the map (Dötsch et al., 2022), the present pattern concerns the items that must be rejected. Both experiments used a single color-defined search task with L and T figures and a fixed target-to-distractor relationship. Whether the same pattern holds for other stimulus dimensions, other target-to-distractor relationships, and other task structures is untested, and we therefore treat this as a candidate extension of the Guided Search framework rather than an established one.

In Experiment 2, out-group pairs searched more efficiently than in-group pairs, even though the display and task were identical. This is the direction predicted by the second of the two accounts set out in the Introduction, on which in-group membership promotes greater self–other overlap (Enock et al., 2020; Aron et al., 2022), rendering partner-related stimuli more self-associated and therefore more resistant to attentional suppression (Sui et al., 2015). It is the opposite of the direction predicted by the co-representation account, on which a partner’s task is taken up more readily for an in-group member (McClung et al., 2013; Müller et al., 2011). We note this correspondence without treating it as decisive, because neither self–other overlap nor co-representation was measured here. This is consistent with a broader pattern in which joint performance is calibrated by how a partner is socially construed, with perceived partner traits (Pan et al., 2026), interpersonal distance, and partner sociality (Zhao et al., 2026), all shown to modulate joint-task performance. Group membership may operate as a similarly consequential social dimension, shaping the difference observed here.

Two considerations qualify this interpretation. First, the group manipulation may have affected processes other than attentional suppression, including perceived similarity, partner novelty, vigilance, discomfort, or demand characteristics. Second, out-group pairs were faster overall and slightly less accurate, raising the possibility that differences in motivation or response strategy contributed to the slope difference. Thus, Experiment 2 supports a descriptive effect of social relationships on joint search performance but does not identify the process underlying this effect.

Several limitations qualify what the present data can support. First, the present sample comprised young university students, and the task relied on artificial stimuli. Future work should test the generality of the effect in more ecologically valid settings and with more diverse participant groups spanning a wider range of ages and with a more balanced sex composition. Second, the search slope provides a clear index of overall efficiency, but it does not identify the process responsible, and neuroimaging and eye-movement measures would be needed to establish whether suppression contributes to it. For example, the Pd component, an electrophysiological marker of active distractor suppression, could test whether joint search elicits stronger suppression of the nontarget items and could distinguish proactive suppression from a learned, safe-to-ignore state in which active inhibition declines (van Moorselaar & Slagter, 2019). Eye-movement measures could likewise test whether joint search reduces fixations on the nontarget items, revealing the contribution of distractor suppression to search guidance (Hamblin-Frohman et al., 2022). Third, Experiment 2 compared two joint conditions without an individual or passive observer baseline, so it establishes a group-related difference in joint search efficiency rather than a difference in the size of any advantage over solitary search. Fourth, the group manipulation was validated by perceived similarity and by the absence of reported suspicion, but identification with the assigned category was not measured. The present data therefore show that the manipulation altered how the partner was construed without establishing how strongly participants identified with their own group. Fifth, the group-related difference in Experiment 2 held across every random-effects structure and both trimming procedures, though on the logarithmic scale it took the form of a constant proportional advantage rather than a difference in per-item cost. We therefore do not present its slope-based form as fully robust, and it awaits independent replication.

## 5. Conclusions

This study shows that searching alongside a partner who is searching the same display for a different target is associated with a shallower search slope than searching alone or alongside a non-searching partner. The slope also differed between in-group and out-group pairs, so this difference in efficiency depends on the social relationship between searchers. On the logarithmic scale this difference took the form of a constant proportional advantage. We interpret these results as consistent with more effective rejection of nontarget items in joint search. The findings also bear on how a co-actor’s task relates to the priority map. Previous work has concentrated on the target side, showing that items a partner searches for gain weight, whereas the present results concern the items a partner must reject. Taken together, these two lines suggest that a subset’s priority in a searcher’s map may depend on the role it plays in the co-actor’s task.

## Figures and Tables

**Table 1:** Fixed-effect estimates from the linear mixed model for condition and set size in Experiment 1.

Variable	Estimate (*β*)	*SE*	*t*	*p*	95% CI
Intercept	729.02	20.22	36.05	<0.001 ***	[689.66, 768.38]
Condition (individual vs. joint)	−13.46	32.41	−0.42	0.679	[−76.56, 49.64]
Condition (individual vs. observer)	33.44	28.60	1.17	0.244	[−22.23, 89.10]
Set size	15.95	0.35	45.67	<0.001 ***	[15.26, 16.63]
Condition (individual vs. joint) × set size	−3.07	0.49	−6.22	<0.001 ***	[−4.04, −2.11]
Condition (individual vs. observer) × set size	−1.16	0.49	−2.35	0.019 *	[−2.13, −0.19]

Note. * *p* < 0.05, *** *p* < 0.001. Reference level: condition = individual. The *p* values are those of the unadjusted model coefficients. The pairwise slope contrasts reported in the text are Tukey-adjusted.

**Table 2:** Fixed-effect estimates from the linear mixed model for condition and set size in Experiment 2.

Variable	Estimate (*β*)	*SE*	*t*	*p*	95% CI
Intercept	759.85	18.35	41.42	<0.001 ***	[724.08, 795.62]
Condition (in-group vs. out-group)	−55.54	25.94	−2.14	0.037 *	[−106.12, −4.95]
Set size	12.67	0.32	39.48	<0.001 ***	[12.04, 13.30]
Condition (in-group vs. out-group) × set size	−1.56	0.45	−3.45	<0.001 ***	[−2.45, −0.67]

Note. * *p* < 0.05, *** *p* < 0.001. Reference level: condition = in-group.

## Data Availability

The repository contains the trial-level data for both experiments, the complete R analysis scripts, which implement the full exclusion pipeline reported here, a variable dictionary for the data files, and the Supplementary Tables and Figures referred to in the text. The MATLAB code used for stimulus generation and presentation is available from the corresponding author on request. All of these materials are available at https://osf.io/vzhca, accessed on 23 August 2026.

## Supplementary Materials

The following supporting information can be downloaded at: https://www.mdpi.com/article/10.3390/bs16091503/s1, Figure S1: Diagnostic plots for the response time models. For each experiment: residuals against fitted values, normal quantile-quantile plot, scale-location plot, and histogram of residuals; Figure S2: Simulated residual diagnostics for the accuracy models, produced with the DHARMa package; Table S1: Trials excluded at each trimming step, by condition and set size; Table S2: Variance components, intraclass correlations, and comparison against a model without the unit level; Table S3: Descriptive response time statistics by condition and set size; Table S4: Complete generalized linear mixed model output for accuracy; Table S5: Fixed-effect estimates from the linear mixed models fitted to log-transformed response times.

## Abbreviations

The following abbreviations are used in this manuscript:

RT	Response time
LMM	Linear mixed model
GLMM	Generalized linear mixed model
RGB	Red–Green–Blue
BFI-2	Big Five Inventory-2
