# Supplementary material for: What Makes Joint Visual Search Efficient? A Distractor Suppression Account
Source: Behav Sci (Basel). 2026 Aug 27;16(9):1503. doi: 10.3390/bs16091503 (PMC13603242; doi:10.3390/bs16091503)
Supplement: Supplementary file 1 [file behavsci-16-01503-s001.zip › behavsci-4481633-supplementary.pdf]

Supplementary Material

What Makes Joint Visual Search Efficient? A Distractor Suppression Account

This file contains the supplementary tables and figures referenced in the manuscript. All analyses were conducted in R 4.6.0; the full scripts are available at https://osf.io/vzhca/overview.

Table S1. Trials excluded at each trimming step, by condition and set size.

Condition	Set Size	Step 1: RT < 200 ms or Error	Step 2: ±2.5 SD Within Participant	Step 3: ±3 SD of the Grand Mean
Experiment 1				
Individual	6	13 (0.95%)	35 (2.58%)	23 (1.74%)
Individual	12	8 (0.58%)	29 (2.13%)	17 (1.28%)
Individual	18	8 (0.58%)	41 (3.01%)	24 (1.82%)
Individual	24	11 (0.80%)	31 (2.28%)	17 (1.28%)
Individual	30	14 (1.02%)	28 (2.07%)	26 (1.96%)
Individual	36	16 (1.17%)	29 (2.14%)	32 (2.42%)
Individual	42	14 (1.02%)	38 (2.81%)	23 (1.75%)
Joint	6	23 (1.68%)	27 (2.01%)	4 (0.30%)
Joint	12	21 (1.54%)	34 (2.52%)	10 (0.76%)
Joint	18	15 (1.10%)	42 (3.10%)	10 (0.76%)
Joint	24	19 (1.39%)	32 (2.37%)	5 (0.38%)
Joint	30	16 (1.17%)	32 (2.37%)	1 (0.08%)
Joint	36	17 (1.24%)	31 (2.29%)	0 (0.00%)
Joint	42	14 (1.02%)	27 (1.99%)	0 (0.00%)
Passive observer	6	14 (1.02%)	31 (2.29%)	13 (0.98%)
Passive observer	12	8 (0.58%)	32 (2.35%)	7 (0.53%)
Passive observer	18	9 (0.66%)	35 (2.58%)	22 (1.66%)
Passive observer	24	15 (1.10%)	34 (2.51%)	6 (0.45%)
Passive observer	30	11 (0.80%)	35 (2.58%)	14 (1.06%)
Passive observer	36	16 (1.17%)	25 (1.85%)	6 (0.45%)
Passive observer	42	5 (0.37%)	36 (2.64%)	9 (0.68%)
Experiment 2				
In-group	6	10 (0.73%)	31 (2.28%)	30 (2.26%)
In-group	12	10 (0.73%)	37 (2.72%)	23 (1.74%)
In-group	18	7 (0.51%)	36 (2.65%)	20 (1.51%)
In-group	24	14 (1.02%)	41 (3.03%)	17 (1.29%)
In-group	30	14 (1.02%)	29 (2.14%)	22 (1.66%)
In-group	36	9 (0.66%)	35 (2.58%)	18 (1.36%)
In-group	42	10 (0.73%)	35 (2.58%)	23 (1.74%)
Out-group	6	25 (1.83%)	33 (2.46%)	1 (0.08%)
Out-group	12	12 (0.88%)	43 (3.17%)	8 (0.61%)
Out-group	18	17 (1.24%)	38 (2.81%)	11 (0.84%)
Out-group	24	10 (0.73%)	32 (2.36%)	10 (0.75%)
Out-group	30	20 (1.46%)	37 (2.74%)	3 (0.23%)
Out-group	36	14 (1.02%)	44 (3.25%)	9 (0.69%)
Out-group	42	11 (0.80%)	40 (2.95%)	7 (0.53%)

Note. Values are the number of trials removed at that step, with the percentage of the trials entering that step in parentheses. Each condition contributed 1,368 trials per set size before trimming. Across all steps, 95.68% of trials were retained in Experiment 1 (95.0% individual, 96.0% joint, 96.0% passive observer) and 95.32% in Experiment 2 (95.1% in-group, 95.6% out-group). No participant lost all of their trials.

Table S2. Variance components, intraclass correlations, and comparison against a model without the unit level.

Panel A. Variance components of the primary response time models

Experiment	Level	Variance	SD	ICC
Experiment 1	Participant within unit	3360.66	57.97	.199
	Unit (dyad)	8839.99	94.02	.524
	Residual	4671.19	68.35	—
Experiment 2	Participant within dyad	4153.85	64.45	.377
	Dyad	2907.44	53.92	.264
	Residual	3947.17	62.83	—

Panel B. Fixed effects under the nested and the participant-only random-effects structures

Experiment	Term	β	SE Nested	p Nested	SE Flat	p Flat
Experiment 1	Intercept	729.02	20.22	<.001	20.19	<.001
	Joint vs. individual	−13.46	32.41	.679	28.56	.638
	Observer vs. individual	33.44	28.60	.244	28.56	.243
	Set size	15.95	0.35	<.001	0.35	<.001
	Joint × set size	−3.07	0.49	<.001	0.49	<.001
	Observer × set size	−1.16	0.49	.019	0.49	.019
Experiment 2	Intercept	759.85	18.35	<.001	16.06	<.001
	Out-group vs. in-group	−55.54	25.94	.037	22.71	.016
	Set size	12.67	0.32	<.001	0.32	<.001
	Out-group × set size	−1.56	0.45	<.001	0.45	<.001

Note. In Experiment 1, a unit is a joint dyad (19 units) or a single participant tested in the individual or passive observer condition (38 units each), giving 95 units for 114 participants. In Experiment 2, every unit is a dyad, giving 38 units for 76 participants. The point estimates are identical to three decimal places under the two random-effects structures; only the standard error of the condition main effect changes. Likelihood-ratio tests favor retaining the unit level in both experiments: Experiment 1 $\chi^2(1) = 11.75$, $p < .001$, AIC 9344.2 versus 9354.0; Experiment 2 $\chi^2(1) = 5.21$, $p = .022$, AIC 6117.0 versus 6120.2.

Table S3. Descriptive response time statistics by condition and set size.

Condition	Set Size	n	M	SD	SE	95% CI
Experiment 1						
Individual	6	38	817	89.6	14.5	[788, 847]
Individual	12	38	933	110.0	17.8	[897, 969]
Individual	18	38	988	118.0	19.1	[950, 1027]
Individual	24	38	1140	143.0	23.2	[1093, 1187]
Individual	30	38	1215	146.0	23.7	[1167, 1263]
Individual	36	38	1297	144.0	23.4	[1249, 1344]
Individual	42	38	1392	150.0	24.3	[1343, 1442]
Joint	6	38	785	89.1	14.5	[756, 814]
Joint	12	38	880	98.3	15.9	[847, 912]
Joint	18	38	922	108.0	17.6	[887, 958]
Joint	24	38	1057	141.0	22.8	[1011, 1103]
Joint	30	38	1098	137.0	22.2	[1054, 1144]
Joint	36	38	1186	144.0	23.3	[1139, 1234]
Joint	42	38	1243	160.0	25.9	[1190, 1295]
Passive observer	6	38	839	94.9	15.4	[807, 870]
Passive observer	12	38	951	118.0	19.1	[912, 989]
Passive observer	18	38	1014	113.0	18.4	[977, 1052]
Passive observer	24	38	1137	133.0	21.6	[1093, 1181]
Passive observer	30	38	1202	150.0	24.4	[1153, 1251]
Passive observer	36	38	1323	145.0	23.5	[1276, 1371]
Passive observer	42	38	1356	136.0	22.1	[1311, 1400]
Experiment 2						
In-group	6	38	821	75.1	12.2	[796, 846]
In-group	12	38	913	92.7	15.0	[882, 944]
In-group	18	38	1000	96.5	15.7	[968, 1031]
In-group	24	38	1064	123.0	20.0	[1023, 1104]
In-group	30	38	1159	120.0	19.4	[1119, 1198]

Condition	Set Size	n	M	SD	SE	95% CI
In-group	36	38	1216	150.0	24.4	[1167, 1266]
In-group	42	38	1276	137.0	22.2	[1230, 1320]
Out-group	6	38	752	58.7	9.5	[733, 772]
Out-group	12	38	834	68.0	11.0	[812, 856]
Out-group	18	38	908	74.0	12.0	[884, 932]
Out-group	24	38	1000	89.6	14.5	[970, 1029]
Out-group	30	38	1048	99.2	16.1	[1016, 1081]
Out-group	36	38	1112	104.0	16.9	[1077, 1146]
Out-group	42	38	1143	119.0	19.3	[1104, 1182]

Note. Response times are in milliseconds and are averaged within participants before aggregation, so n is the number of participants contributing to each cell. Confidence intervals are based on the t distribution.

Table S4. Complete generalized linear mixed model output for accuracy.

Experiment	Term	β (log-odds)	SE	z	p	95% CI
Experiment 1	Intercept	5.175	0.321	15.63	<.001	[4.545, 5.805]
	Joint vs. individual	−0.897	0.412	−2.18	.030	[−1.706, −0.089]
	Observer vs. individual	0.053	0.455	0.12	.908	[−0.840, 0.945]
	Set size	0.007	0.011	0.65	.514	[−0.014, 0.028]
	Joint × set size	0.008	0.013	0.58	.562	[−0.019, 0.034]
	Observer × set size	0.000	0.015	0.01	.995	[−0.030, 0.030]
Experiment 2	Intercept	5.414	0.346	15.63	<.001	[4.735, 6.093]
	Out-group vs. in-group	−1.048	0.442	−2.37	.018	[−1.915, −0.181]
	Set size	−0.004	0.011	−0.41	.684	[−0.025, 0.016]
	Out-group × set size	0.020	0.014	1.48	.138	[−0.006, 0.047]

Note. Models were fitted with a binomial link and random intercepts for participants nested within units, matching the response time models. Confidence intervals are Wald intervals. Adding random slopes for set size did not improve either model, Experiment 1 $\chi^2(4) = 0.57$, $p = .966$; Experiment 2 $\chi^2(4) = 1.56$, $p = .817$.

Table S5. Fixed-effect estimates from the linear mixed models fitted to log-transformed response times.

Experiment	Term	β	SE	df	t	p
Experiment 1	Intercept	6.6398	0.0184	145.57	361.17	<.001
	Joint vs. individual	−0.0330	0.0294	107.41	−1.12	.265
	Observer vs. individual	0.0338	0.0260	145.57	1.30	.195
	Set size	0.0147	0.0003	681.00	45.76	<.001
	Joint × set size	−0.0019	0.0005	681.00	−4.25	<.001
	Observer × set size	−0.0011	0.0005	681.00	−2.46	.014
Experiment 2	Intercept	6.6634	0.0176	53.83	378.59	<.001
	Out-group vs. in-group	−0.0793	0.0249	53.83	−3.19	.002
	Set size	0.0121	0.0003	454.00	38.45	<.001
	Out-group × set size	−0.0004	0.0004	454.00	−0.95	.342

Note. Response times were log-transformed before fitting; the random-effects structure and the trimming procedure were identical to those of the primary models. Coefficients are on the log scale, so a condition effect of β corresponds to a multiplicative change of exp(β) in response time at every set size. In Experiment 1, the condition-by-set-size interaction remained significant after transformation. In Experiment 2, the interaction was not significant, whereas the main effect of condition was, which corresponds to a constant proportional advantage of 7.6% for out-group pairs across set sizes.

Model diagnostics and influential cases

Figure S1. Diagnostic plots for the response time models. For each experiment: residuals against fitted values, normal quantile–quantile plot, scale–location plot, and histogram of residuals.

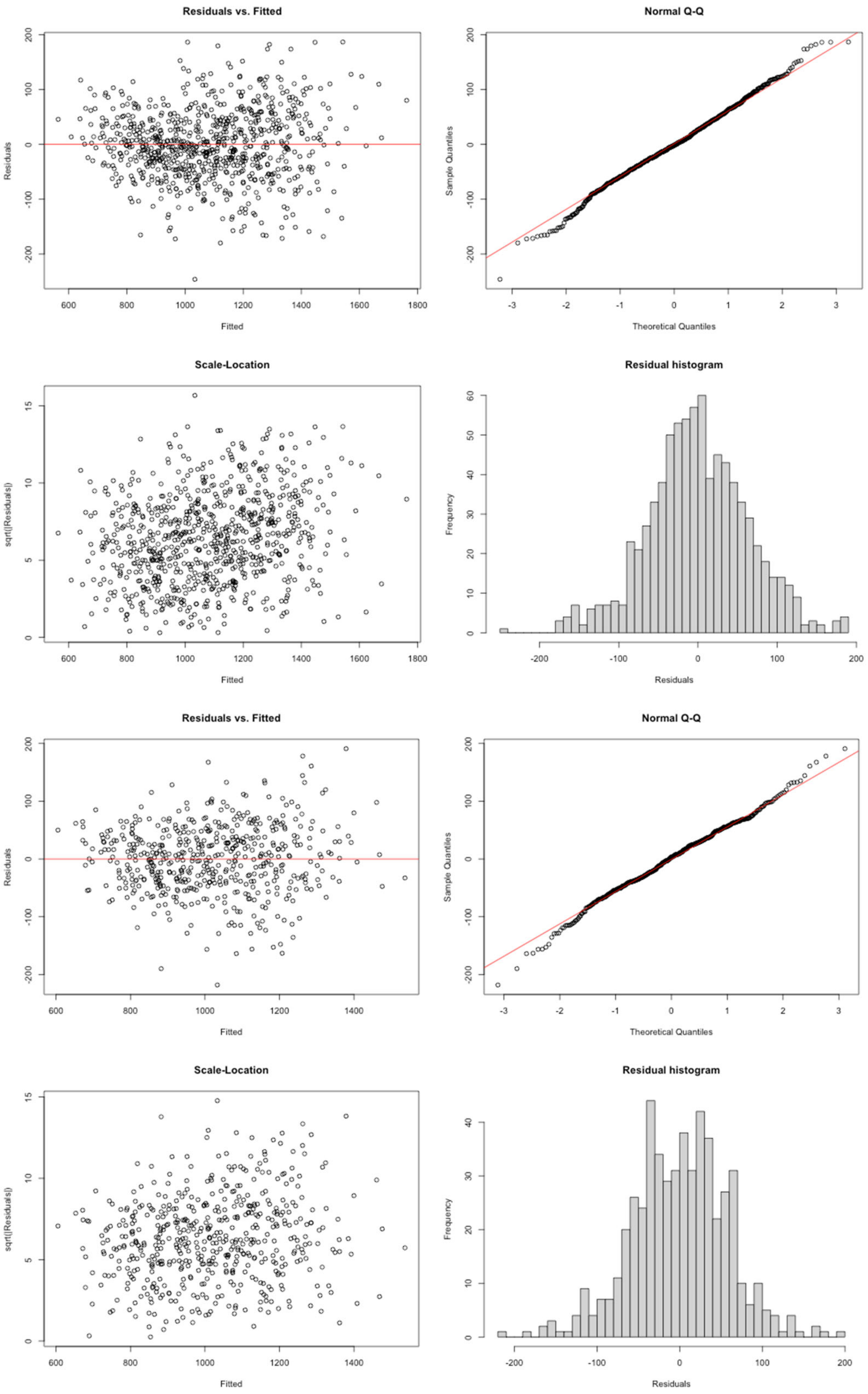

Figure S2. Simulated residual diagnostics for the accuracy models, produced with the DHARMa package.

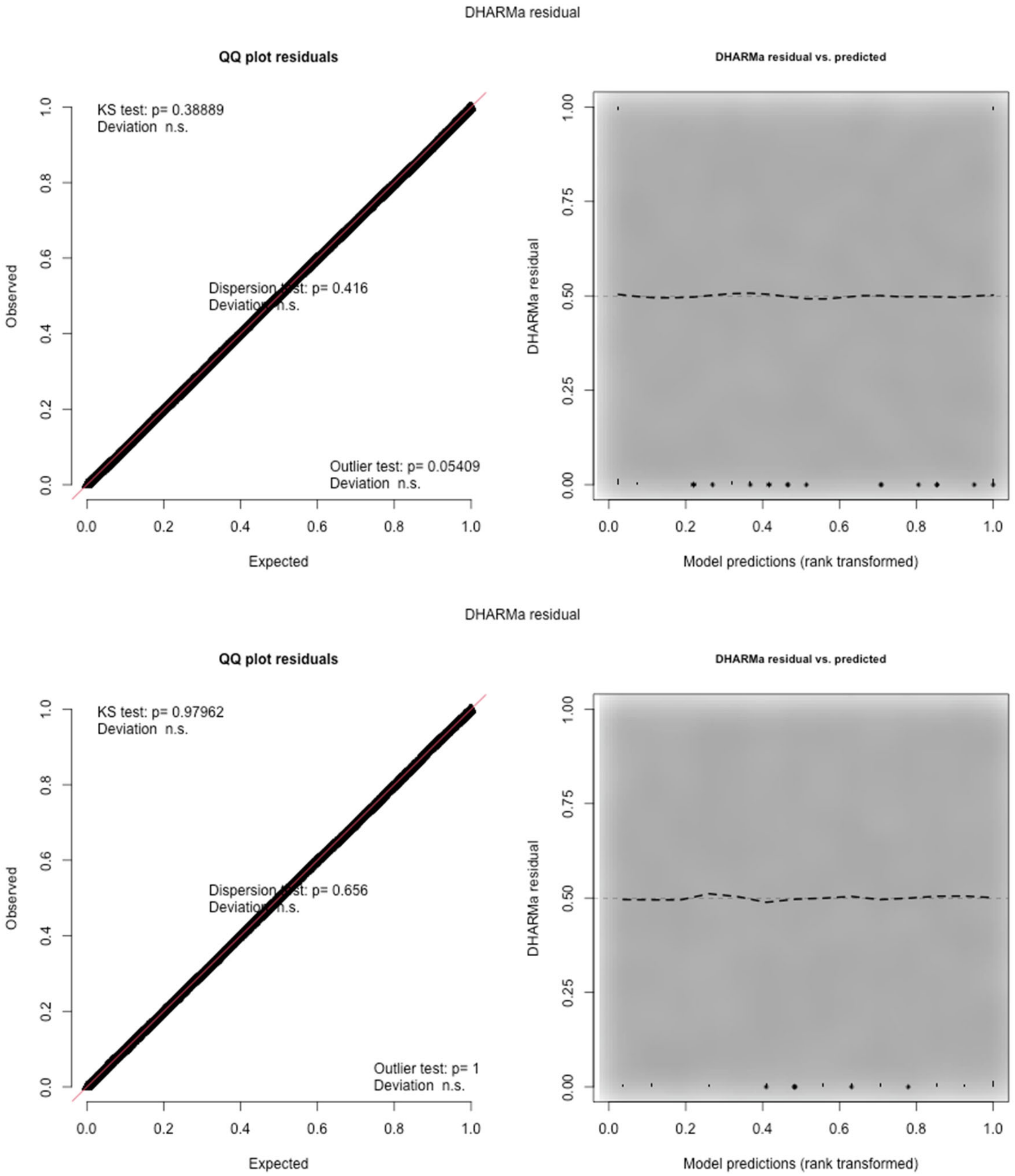

Influential cases

Influence was assessed with Cook's distance computed at the level of the independent unit, using the conventional threshold of 4/n.

In Experiment 1, eight of the 95 units exceeded the threshold of .042 (units 6, 15, 16, 17, 1005, 1016, 1022, and 2012). Refitting without them retained 87 units and 714 observations. The effects were attenuated but not removed: the joint versus individual slope difference fell from 3.07 to 2.19 ms per item, SE = 0.50, $t(609) = 4.35$, $p < .001$, and the joint versus passive observer difference fell from 1.91 to 1.50 ms per item, SE = 0.50, $t(609) = 3.03$, $p = .007$, while the non-focal comparison between the passive observer and individual conditions fell from 1.16 to 0.68 ms per item, $p = .324$.

In Experiment 2, six of the 38 dyads exceeded the threshold of .105 (dyads 4, 8, 1005, 1011, 1013, and 1014). Refitting without them retained 32 dyads and 448 observations and reduced the condition by set size interaction from −1.56 to −1.13 ms per item while leaving it significant, SE = 0.48, $t(382) = -2.36$, $p = .019$. The main

conclusions are therefore unchanged by the exclusion of these units in either experiment.

Simplified random-slope structures

Because the two-level random-slope model produced a singular fit in both experiments, two simplified structures were fitted: one retaining slopes at both levels but omitting the intercept–slope correlations, and one retaining slopes at the participant level only with the unit level as a random intercept. Both converged without a singular fit in either experiment.

In Experiment 1, AIC was 9280.3 for the uncorrelated structure and 9280.4 for the participant-slopes structure, against 9344.2 for the random-intercept model and 9281.5 for the full random-slope model. The joint versus individual slope difference was −3.07 ms per item in both, $t(70.43) = -4.16$ and $t(111) = -4.48$, both $p < .001$, and the joint versus passive observer difference was −1.91 ms per item, $p = .031$ and $p = .017$, respectively. The non-focal comparison between the passive observer and individual conditions was not significant under either structure, $p = .230$ and $p = .212$.

In Experiment 2, AIC was 6068.1 and 6068.7 for the two simplified structures, against 6117.0 for the random-intercept model and 6067.4 for the full random-slope model. The condition by set size interaction was −1.56 ms per item in both, $t(39.67) = -2.16$, $p = .037$ and $t(74) = -2.48$, $p = .016$.

Response order and partner-response dependency

We performed a further analysis to address the possibility that the joint advantage reflects coordination between partners rather than a difference in search efficiency. A pair of responses was retained only when both members survived trimming, leaving 4,547 paired trials in Experiment 1 and 8,884 in Experiment 2.

A partner's response time on a given trial predicted a participant's own response time, but weakly: $\beta = 0.072$, $SE = 0.010$, $p < .001$ in Experiment 1, so a partner 1,000 ms slower was associated with the participant being 72 ms slower. Covariation of this size is expected from shared display difficulty alone.

Responses were classified as first or second by which member responded earlier on each trial; across participants, the proportion of trials on which a participant responded first averaged .50 (range .29 to .71). In Experiment 1, the search slope was 8.68 ms/item for first responses and 16.93 ms/item for second responses; difference = 8.25, $p < .001$. The interval between the two responses grew with set size by 8.23 ms per item; $SE = 0.36$, $p < .001$, rather than shrinking as convergence on a common response time would predict.

These two subsets are not random halves of the joint condition. Classifying responses by which member responded earlier takes the faster of two draws on each trial for the first-response subset and the slower for the second-response subset, and because response-time variability grows with set size, so does the separation this produces. The two subsets should therefore fall symmetrically about the joint slope, at a distance of about half the rate at which the interval between responses grows. They do: the joint slope was 12.87 ms/item, and the two subsets lay 4.19 below and 4.06 above it, against a predicted displacement of 4.12. Coordination would compress the second response toward the first, particularly at larger set sizes where the two searchers are furthest apart, and would therefore make these displacements asymmetric. No such asymmetry was present. For completeness, the slope for first responses in Experiment 1 was 8.67 ms/item against 15.93 ms/item in the individual condition, and the slope for second responses was 16.93 ms/item; the first-response comparison is inflated by the selection just described and should not be read as an estimate of the joint advantage, which remains the 3.07 ms/item reported in the main text. In Experiment 2, where both conditions are subject to the same selection, the group difference held within both subsets, with slopes of 8.12 against 7.07 ms/item for first responses, difference = 1.05, $p = .005$, and 16.7 against 15.0 ms/item for second responses, difference = 1.75, $p = .002$.

Convergence

All primary random-intercept models converged without warnings and were not singular. Both random-slope models produced singular fits, with the unit-level intercept and slope correlation estimated at 1.00, which is why the random-intercept specification is retained as primary and the random-slope results are reported as robustness checks. The random-slope accuracy models were also singular and did not improve fit.
